# Association Between Serum Uric Acid and Carotid Intima-Media Thickness in Different Fasting Blood Glucose Patterns: A Case-Control Study

**DOI:** 10.3389/fendo.2022.899241

**Published:** 2022-05-27

**Authors:** Yuanyuan Gao, Baofeng Xu, Yanyan Yang, Mei Zhang, Tian Yu, Qiujuan Zhang, Jianwei Sun, Rui Liu

**Affiliations:** ^1^ Department of VIP Unit, China-Japan Union Hospital of Jilin University, Changchun, China; ^2^ Department of Endocrinology, China-Japan Union Hospital of Jilin University, Changchun, China; ^3^ Department of Stroke Center, First Hospital of Jilin University, Changchun, China; ^4^ Department of Ultrasound, China-Japan Union Hospital of Jilin University, Changchun, China; ^5^ Department of Neurosurgery, Weifang People’s Hospital, Weifang, China

**Keywords:** serum uric acid, carotid atherosclerosis, carotid intima-media thickness, fasting blood glucose, case-control study

## Abstract

Our objective was to analyze the correlation between serum uric acid (SUA) levels and carotid intima-media thickness (CIMT) and explore the relationship between SUA and carotid atherosclerosis in different glucose metabolism patterns. A total of 614 patients were enrolled in this case-control study, including 406 in the normouricemia group and 208 in the hyperuricemia group. The two groups were each divided into three groups according to fasting blood glucose (FBG) level: normal, impaired fasting glucose (IFG), and diabetes mellitus (DM). CIMT and the CIMT thickening rate in the hyperuricemia group were significantly higher than those in the normouricemia group: 0.17 (0.11–0.24) cm vs. 0.12 (0.08–0.15) cm and 73.56% vs. 51.97% (*p* < 0.001). Pearson’s correlation analysis showed that age, systolic blood pressure (SBP), diastolic blood pressure, FBG, triglyceride, SUA, creatinine, and blood urea nitrogen were positively correlated with CIMT, whereas high-density lipoprotein cholesterol and total cholesterol were negatively correlated with CIMT. Multiple linear regression analysis showed that age, SUA, FBG, and SBP were independent factors that affected CIMT. Furthermore, age and SBP were independent factors in the normouricemia group, and FBG was an independent factor that affected CIMT in the hyperuricemia group (*p* < 0.05). In the hyperuricemia group, CIMT in the DM group was significantly higher than that in the normal group [0.20 (0.14–0.25)cm vs. 0.15 (0.1–0.25); *p* < 0.05], and the CIMT thickening rate in the DM group was significantly higher than those in the IFG and normal groups (90.38% vs. 78.38%, 90.38% vs. 65.81%; *p* < 0.05). The ROC curve analysis showed that uric acid combined with age, SBP, and FBG had the highest area under the curve (AUC) for predicting CIMT thickening [0.855 (95% confidence interval (CI): 0.804–0.906)], followed by uric acid combined with FBG [AUC: 0.767 (95% CI: 0.726–0.808)]. In conclusion, SUA was closely associated with an increase in CIMT in patients with specific FBG metabolic patterns and may be an independent risk factor for carotid atherosclerosis. SUA, especially in combination with other factors (such as age, SBP, FBG), may serve as a specific model to help predict the incidence of CIMT thickening. **Clinical Trial Registration:** http://www.chictr.org.cn, identifier ChiCTR2000039124.

## Introduction

Stroke is currently the second leading cause of death worldwide, accounting for 11.8% of all deaths (1). In China, cardiovascular and cerebrovascular diseases are the most common causes of death, and there are approximately 250 million new stroke patients each year (2, 3). With the growing older adult population, the incidence of such events will likely continue to increase, most of which will be ischemic stroke (4). Carotid atherosclerosis plays an important role in the development of stroke, 18%–25% of ischemic strokes are attributed to thromboembolism due to carotid atherosclerotic disease (5–7). Approximately 28% of the global general population aged 30–79 years had an intima-media thickness abnormality of 1.0 mm or greater in 2020, which suggests that the number of people with an intima-media thickness abnormality could be as high as 1 billion (8). In China, the number of people affected by carotid atherosclerosis and carotid plaques was subsequently anticipated to reach 267.25 million and 199.83 million, respectively, by 2020 (9). Early identification of risk factors and diagnosis of carotid vascular diseases are clinically significant for the prevention of stroke. The carotid artery provides a “window” that reflects systemic arteriosclerosis, which is directly related to the development of cerebrovascular disease (10). CIMT measured using arterial ultrasound is a common clinical indicator of the degree of atherosclerosis, and increased CIMT is significantly associated with an increased risk of stroke (10). Thus, CIMT can be used to clinically assess the overall risk of stroke (11).

Hyperuricemia is a metabolic disease caused by purine metabolism or uric acid excretion disorders. Elevated uric acid levels are associated with numerous recognized risk factors for cardiovascular disease, which include age, hypertension, hyperlipidemia, obesity, and diabetes (12–16). The prevalence of hyperuricemia (serum uric acid [SUA] level > 7 mg/dl) in the adult population in the United States is 11.9% based on the United States National Health and Nutrition Examination Survey (17). A recent study showed that the incidence of hyperuricemia in China is 18.4% (18). Since the 1950s, the correlation between uric acid and cardiovascular and cerebrovascular diseases has continued to attract considerable attention (19). Similarly, several studies reported that SUA levels were independently associated with the development of cardiovascular and cerebrovascular diseases, especially myocardial infarction and stroke; therefore, SUA is a strong predictor of subsequent cardiovascular diseases and all-cause death (20–23). The result of a recent meta-analysis showed that patients with hyperuricemia had a higher incidence of CIMT thickening than the normal population (24).

Hyperuricemia is typically accompanied by a state of hyperinsulinemia or diabetes mellitus (DM); abnormal glucose metabolism is accompanied by abnormal renal function, which results in impaired excretion of uric acid in the body and ultimately hyperuricemia (25). A study in a Chinese population showed that fasting blood glucose (FBG) was an independent risk factor for CIMT, whereby CIMT levels increase significantly with changes in glucose metabolism (26). However, there have not been any studies examining whether the relationship between SUA and CIMT is consistent under different patterns of glucose metabolism. Therefore, we conducted this clinical study to explore the relationship between SUA and carotid atherosclerosis under different glucose metabolic conditions toward the early detection of risk factors and the active prevention of strokes.

## Materials and Methods

### Inclusion and Exclusion Criteria

From October 1, 2020, to November 20, 2021, medical histories and examination results of inpatients were obtained from the China-Japan United Hospital of Jilin University and the First Hospital of Jilin University. Inclusion criteria were as follows: 1) ability to understand and willingness to provide written informed consent and 2) aged over 18 years. Exclusion criteria were as follows: 1) severely impaired liver and kidney function, 2) use of diuretics in the past one month, 3) history of malignant tumors or received radiotherapy within the past 3 months, 4) acute stroke, 5) autoimmune diseases, 6) blood system diseases, 7) development of a serious infection in the past month, 8) acute complications of diabetes, 9) cognitive disorders, 10) acute respiratory failure, 11) acute myocardial infarction, and 12) administered antituberculous drugs within the past month. The study was approved by the Ethics Committee of the China-Japan Union Hospital of Jilin University (2020032509) and the Ethics Committee of the First Hospital of Jilin University (2020-420). The clinical trial registration number is ChiCTR2000039124.

### Clinical and Laboratory Evaluation

Basic information, such as gender, age, height, weight, and blood pressure were collected, and body mass index (BMI) was calculated as weight (kg) divided by height squared (m^2^). All patients fasted for at least 10 hours. Blood and urine were collected the following morning to assess FBG, triglyceride (TG), total cholesterol (TC), low-density lipoprotein cholesterol (LDL-C), high-density lipoprotein cholesterol (HDL-C), SUA, creatinine (Cr), blood urea nitrogen (BUN), and urine pH levels. SUA levels were detected using the uricase-peroxidase method with a Beckman Coulter biochemical analysis system (Beckman Coulter Inc., Brea, CA, USA). Patients were divided into normouricemia and hyperuricemia groups according to the SUA cut off level of 420 μmol/L (27). A total of 614 patients participated in the case-control study, including 406 (66.12%) in the normouricemia group and 208 (33.88%) in the hyperuricemia group. All subjects were aged between 22 and 89 years. The proportions of males in the normouricemia and hyperuricemia groups were 48.52% and 84.13%, respectively. Patients in the normouricemia and hyperuricemia groups were each divided into three groups according to FBG levels: normal (FBG < 6.1 mmol/L; n = 205 and 117, respectively), impaired fasting glucose (IFG; FBG 6.1–7.0 mmol/L; n = 45 and 37, respectively), and DM (FBG ≥ 7.0 mmol/L or used antidiabetic drugs in patients with diabetes) (28) (n = 150 and 52, respectively). Eight patients were excluded from the blood glucose subgroup analysis due to missing FBG values.

### Evaluation of Carotid Atherosclerosis

Experienced physicians used high-resolution real-time B-ultrasound (Philips EPIQ 7; Philips, Amsterdam, Netherlands) to examine the neck vessels. CIMT measurements were performed 10 mm proximal to the bifurcation of both carotid arteries. In the absence of plaques, the thickest CIMT was used. If plaques were present, the maximum thickness diameter of the plaque was used for statistical analysis. Measurements were taken three times, and an average value was obtained. CIMT < 1.0 mm was defined as normal intima, CIMT ≥ 1.0 mm was regarded as thickened, and localized CIMT ≥ 1.5 mm was defined as a plaque (8, 29). The CIMT thickening rate is defined as the percentage of the population with CIMT ≥ 1.0 mm.

### Statistical Analysis

Statistical analysis was performed using SPSS 25.0. Quantitative variables were described as means ± standard deviations or medians (interquartile ranges). T-tests and rank-sum tests were used to compare groups. Pearson’s correlation was used for correlation analysis. Using CIMT as the dependent variable, a multiple linear regression model was used to adjust for influencing factors and evaluate the relationship between SUA and CIMT. The area under the receiver operating characteristic (ROC) curve (AUC) indicated the specificity of the risk factors in predicting CIMT thickening. If necessary, logarithms were used for statistical correlation analysis. A *p* < 0.05 was considered statistically significant.

## Results

### Basic Characteristics of the Population

**Figure 1:**
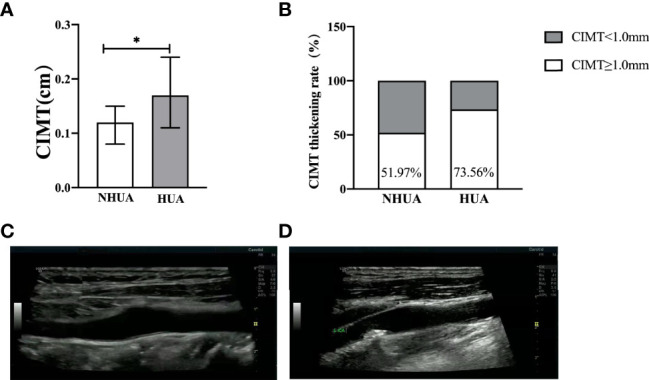
**(A, B)** Comparison of CIMT and CIMT thickening rate between the normouricemia and hyperuricemia groups. CIMT and the CIMT thickening rate in the hyperuricemia group were significantly higher than those in the normouricemia group (*p* < 0.001). **(C, D)** The B-ultrasound image of the normal carotid intima-media thickness and carotid intima-media thickness thickening. NHUA: normouricemia group (n = 406); HUA, hyperuricemia group (n = 208). *p < 0.05.

In the normouricemia group, there were significant differences under different FBG metabolism patterns in gender, age, BMI, systolic blood pressure (SBP), TG, LDL-C, HDL-C, Cr, and BUN (*p* < 0.05), whereas there were no significant differences in diastolic blood pressure (DBP) and TC (*p >*0.05). In the hyperuricemia group, there were differences under different FBG metabolism in gender and TG level (*p* < 0.05) but not in age, BMI, SBP, DBP, TC, LDL-C, HDL-C, Cr, and BUN levels (*p* > 0.05; **Table 1**). CIMT and the CIMT thickening rate in the hyperuricemia group were significantly higher than those in the normouricemia group: 0.17 (0.11–0.24) cm vs. 0.12 (0.08–0.15) cm and 73.56% vs. 51.97% (*p* < 0.001), respectively (**Figure 1**).

**Table 1:** Characteristics of the study population.

	Normouricemic group (n=400)				*p* value	Hyperuricemic group (n=206)				*P* value
	Total (n=400)	Nomal (n=205)	IFG (n=45)	DM (n=150)		Total (n=206)	Nomal (n=117)	IFG (n=37)	DM (n=52)	
Age (years)	55.00 (44.00,64.00)	50.00 (38.00,62.00)	61.00 (55.00,67.00)	57.00 (51.00,65.00)	<0.05	56.00 (46.00,64.75)	56.00 (43.00,64.00)	58.00 (48.00,65.00)	55.00 (50.00,63.75)	> 0.05
Male%	48.52	41.46	40.00	58.00	<0.05	84.13	87.18	78.38	80.77	<0.05
BMI (kg/m2)	24.45 (22.70,26.96)	23.20 (21.30,25.93)	24.80 (23.10,27.03)	25.10 (23.68,27.83)	<0.05	25.40 (23.70,27.70)	25.40 (23.38,27.30)	25.45 (23.85,27.95)	26.55 (23.68,28.48)	> 0.05
SBP (mmHg)	133.00 (121.00,146.00)	138.00 (114.00,142,00)	142.00 (130.75,150.25)	135.00 (126.00,146.00)	<0.05	139.00 (128.00,150.00)	135.00 (128.00,145.00)	143.00 (130.00,155.50)	137.00 (126.50,152.00)	> 0.05
DBP (mmHg)	80.00 (70.00,88.00)	79.00 (69.00,86.00)	84.00 (74.75,90.00)	80.00 (72.00,87.00)	> 0.05	82.00 (77.00,93.00)	80.00 (76.00,90.00)	85.00 (80.00,94.50)	82.00 (79.50,96.00)	> 0.05
FBG (mmol/L)	5.97 (5.12,8.30)	5.15 (4.77,5.45)	6.64 (6.46,6.85)	9.38 (7.89,11.92)	<0.05	5.80 (5.12,7.02)	5.24 (4.87,5.60)	6.50 (6.27,6.63)	8.54 (7.70,10.89)	<0.05
TG (mmol/L)	1.51 (1.09,2.20)	1.30 (0.95,1.78)	1.89 (1.13,2.43)	1.72 (1.33,2.54)	<0.05	2.18 (1.46,3.55)	1.89 (1.41,3.01)	2.39 (1.57,3.93)	2.80 (1.95,4.13)	<0.05
TC (mmol/L)	5.10 (4.28,5.92)	5.02 (4.34,5.84)	5.20 (4.10,6.08)	5.16 (4.37,6.06)	> 0.05	5.32 (4.55,6.01)	5.29 (4.55,5.85)	5.37 (4.35,6.54)	5.26 (4.80,6.12)	> 0.05
HDL-C (mmol/L)	1.23 (1.04,1.46)	1.33 (1.14,1.55)	1.18 (0.99,1.46)	1.10 (0.98,1.30)	<0.05	1.09 (0.93,1.26)	1.11 (0.97.1.27)	1.82 (0.93,1.26)	2.19 (0.90,1.22)	> 0.05
LDL-C (mmol/L)	3.00 (2.37,3.56)	2.76 (2.32,3.35)	3.08 (2.33,3.79)	3.11 (2.53,3.76)	<0.05	3.15 (2.68,3.67)	3.04 (2.66,3.51)	3.38 (2.69,4.10)	3.23 (2.72,3.71)	> 0.05
Cr (umol/L)	65.40 (57.86,76.62)	66.51 (60.25,79.84)	67.61 (57.80,78.79)	62.22 (54.49,71.62)	<0.05	80.70 (67.92,93.41)	80.70 (67.93,93.19)	79.68 (65.60,87.51)	79.30 (65.18,91.55)	> 0.05
BUN (umol/L)	5.16 (4.30,5.94)	4.88 (4.02,5.69)	5.61 (4.65,6.69)	5.41 (4.69,6.33)	<0.05	5.65 (4.66,6.89)	5.40 (4.56,6.31)	6.02 (5.40,6.75)	5.90 (4.72,7.42)	> 0.05

BMI, body mass index; SBP, systolic blood pressure; DBP, diastolic blood pressure; FBG, fasting blood glucose; TG, triglycerides; TC, cholesterol; LDL-C, low-density lipoprotein cholesterol; HDL-C, high-density lipoprotein cholesterol; BUN, blood urea nitrogen; Cr, creatinine. Statistical methods: Chi-square test was used for gender comparison between groups. Age, BMI, SBP, DBP, FBG, TG, TC, HDL-C, LDL-C, Cr, and BUN were compared between groups by rank-sum test.

### SUA as an Independent Influencing Factor of CIMT

Pearson correlation analysis showed that age (r = 0.552, *p* < 0.001), SBP (r = 0.317, *p* < 0.001), DBP (r = 0.195, *p* < 0.001), FBG (r = 0.250, *p* < 0.001), TG (r = 0.177, *p* < 0.001), SUA (r = 0.255, *p* < 0.001), Cr (r = 0.146, *p* < 0.001), and BUN (r = 0.265, *p* < 0.001) were positively correlated with CIMT, whereas HDL-C (r = −0.245, *p* < 0.001) and TC (r = −0.094, *p* = 0.021) were negatively correlated with CIMT (**Table 2**). Age had the strongest correlation with CIMT, followed by SBP, SUA, and FBG.

**Table 2:** Correlation analysis of CIMT with general data and biochemical indexes.

	r	*P* value
Age	0.552	<0.001
BMI	0.073	0.109
SBP	0.317	<0.001
DBP	0.195	<0.001
SUA	0.255	<0.001
FBG	0.250	<0.001
U-PH	-0.550	0.182
TG	0.177	<0.001
TC	-0.094	0.021
HDL-C	-0.245	<0.001
LDL-C	0.045	0.263
Cr	0.146	<0.001
BUN	0.265	<0.001

Pearson correlation analysis showed that age (r = 0.552, p < 0.001), SBP (r = 0.317, p < 0.001), DBP (r = 0.195, p < 0.001), FBG (r = 0.250, p < 0.001), TG (r = 0.177, p < 0.001), SUA (r = 0.255, p < 0.001), Cr (r = 0.146, p < 0.001), and BUN (r = 0.265, p < 0.001) were positively correlated with CIMT, whereas HDL-C (r = −0.245, p < 0.001) and TC (r = −0.094, p = 0.021) were negatively correlated with CIMT.

Multivariate linear regression analysis showed that after adjusting for age, SBP, DBP, SUA, FBG, TG, TC, HDL-C, Cr, and BUN levels, the factors that independently influenced CIMT were age [β = 0.37, 95% confidence interval (CI): 0.62–0.98, *p* < 0.001], SUA (β = 0.25, 95% CI: 0.25–0.55, *p* < 0.001), SBP (β = 0.15, 95% CI: 0.14–0.86, *p* = 0.007), and FBG (β = 0.13, 95% CI: 0.07–0.30, *p* = 0.002). Age and SUA were the most independent (**Table 3**).

**Table 3:** Multiple linear regression analysis of CIMT as dependent variable.

	β	SE	β(95%CI)	*P* value
Age	0.37	0.09	(0.62,0.98)	<0.001
SUA	0.25	0.08	(0.25,0.55)	<0.001
SBP	0.15	0.19	(0.14,0.86)	0.007
FBG	0.13	0.06	(0.07,0.30)	0.002

Take CIMT as the dependent variable, multivariate linear regression analysis showed that after adjusting for age, SBP, DBP, SUA, FBG, TG, TC, HDL-C, Cr, and BUN levels, the factors that independently influenced CIMT were age (β = 0.37, 95% CI: 0.62–0.98, p < 0.001), SUA (β = 0.25, 95% CI: 0.25–0.55, p < 0.001), SBP (β = 0.15, 95% CI: 0.14–0.86, p = 0.007), and FBG (β = 0.13, 95% CI: 0.07–0.30, p = 0.002).

### SUA and CIMT in Different FBG Patterns

**Figure 2:**
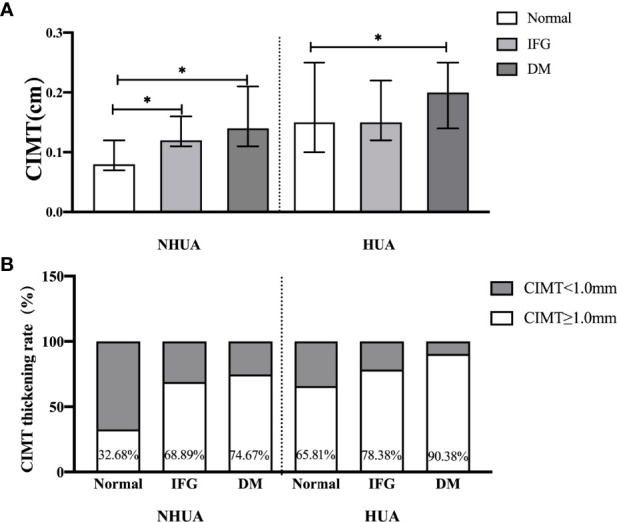
CIMT and CIMT thickening rate for different FBG metabolism patterns. Patients were divided into three groups according to fasting blood glucose metabolism patterns: normal, impaired fasting glucose (IFG), and diabetes mellitus (DM). Under different FBG metabolism patterns, CIMT and the CIMT thickening rate in the hyperuricemia group were higher than those in the normouricemia group (*p* < 0.05). CIMT and CIMT thickening rate in the hyperuricemia group showed a trend gradual increase with the increase in FBG (*p* < 0.05). NHUA, normouricemia group (Normal, n=205; IFG, n=45; DM, n=150); HUA, hyperuricemia group (Normal, n=117; IFG, n=37; DM, n=52). *p < 0.05.

Multivariate analysis showed that age (*p* < 0.001) and SBP (*p* = 0.041) were independent risk factors for CIMT in the normouricemia group, whereas FBG was an independent risk factor for CIMT in the hyperuricemia group (*p* = 0.033; **Table 4**). In the normal, IFG, and DM groups, CIMTs in the hyperuricemia group were significantly higher than that in the normouricemia group: 0.15 (0.10–0.25) cm vs. 0.08 (0.07–0.12) cm, 0.15 (0.12–0.22) cm vs. 0.12 (0.11–0.16) cm, and 0.20 (0.14–0.25) cm vs. 0.14 (0.11–0.21) cm (*p* < 0.05), respectively (**Figure 2A**). Similarly, the CIMT thickening rates in the hyperuricemia group were significantly higher than that in the normouricemia group across all three FBG groups: 65.81% vs. 32.68%, 78.38% vs. 68.89%, and 90.38% vs. 74.67% (*p* < 0.05), respectively (**Figure 2B**). In the hyperuricemia group, results showed that CIMT in the DM group was significantly higher than that in the normal group [0.2 (0.14–0.25) cm vs. 0.15 (0.1–0.25); *p* < 0.05; **Figure 2A**], and the CIMT thickening rate in the DM group was significantly higher than that in the IFG and normal groups (90.38% vs. 78.38% and 90.38% vs. 65.81%, respectively; *p* < 0.05; **Figure 2B**). Under different FBG metabolism patterns, CIMT and the CIMT thickening rate in the hyperuricemia group were higher than those in the normouricemia group. Moreover, CIMT and the CIMT thickening rate in the hyperuricemia group showed a trend gradual increase with the increase in FBG.

**Table 4:** Determinants of traditional risk factors for CIMT in different SUA groups in a multivariate analysis.

	Normouricemic group				Hyperuricemic group			
	β	SE	(95%CI)	*P* value	β	SE	(95%CI)	*P* value
Age	0.35	0.12	(0.48,0.95)	<0.001	–	–	–	–
BMI	0.05	0.19	(-0.21,0.53)	0.383	–	–	–	–
SBP	0.12	0.19	(0.02,0.74)	0.041	–	–	–	–
FBG	0.09	0.07	(0.25,0.80)	0.148	0.156	0.13	(0.02,0.54)	0.033
TG	-0.06	0.05	(-0.15,0.05)	0.349	0.01	0.06	(-0.12,0.13)	0.905
HDL-C	-0.06	0.11	(-0.32,0.12)	0.360	–	–	–	–
LDL-C	-0.02	0.08	(-0.18,0.14)	0.790	–	–	–	–
Cr	0.03	0.08	(-0.11,0.19)	0.623	–	–	–	–
BUN	0.03	0.09	(-0.14,0.21)	0.672	–	–	–	–

Take CIMT as the dependent variable, multivariate analysis showed that age (p < 0.001) and SBP (p = 0.041) were independent risk factors for CIMT in the normouricemia group, whereas FBG (p = 0.033) was an independent risk factor for CIMT in the hyperuricemia group.

### SUA Combined With Age, FBG, and SBP as the Best Index for Diagnosing CIMT Thickening

**Figure 3:**
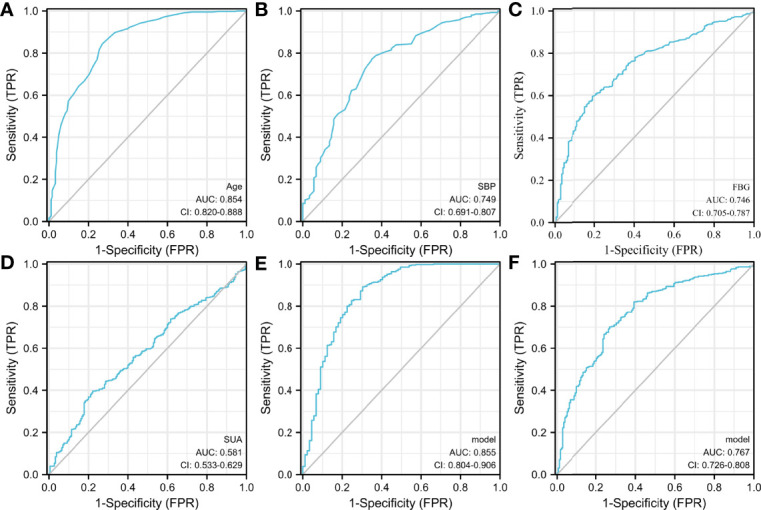
The receiver operating characteristic (ROC) curve. **(A–D)** Specificity of age, SBP, FBG, and SUA for predicting the thickening of CIMT. **(E)** Specificity of SUA combining age, SBP, and FBG for predicting the thickening of CIMT. **(F)** Specificity of SUA combined with FBG for predicting the thickening of CIMT. SUA combined with age, SBP, and FBG had the highest AUC for predicting the thickening of CIMT, followed by SUA combined with FBG.

The ROC curve analysis showed that the AUCs for using age, SBP, FBG, and SUA to predict CIMT were 0.854 (95% CI: 0.820–0.888), 0.749 (95% CI: 0.691–0.807), 0.746 (95% CI: 0.705–0.787), and 0.581 (95% CI: 0.533–0.629), respectively (**Figures 3A–D**). SUA combined with age, SBP, and FBG had the highest AUC for predicting the thickening of CIMT [AUC: 0.855 (95% CI: 0.804–0.906)] (**Figure 3E**), followed by SUA combined with FBG [AUC: 0.767 (95% CI: 0.726–0.808); **Figure 3F**]. Age was the most significant factor, and the inclusion of factors such as SBP, FBG, and SUA helped improve diagnostic efficiency.

## Discussion

In this case-control study, we evaluated the relationship between SUA and CIMT in the Chinese population and the correlation between SUA and CIMT in different glucose metabolism states. We found that patients with high uric acid had higher CIMT and CIMT thickening rates than those with normal uric acid. Meanwhile, CIMT and the CIMT thickening rate in the hyperuricemia group showed a trend of gradual increase with the increase in FBG. By examining cross-sectional data of the population, there was a significant association between SUA and CIMT, which persisted even after adjusting for traditional confounding factors, such as age, blood pressure, lipids, and FBG. Serum uric acid can be used as an independent risk factor for CIMT thickening. Furthermore, in addition to uric acid level, age, SBP, and FBG were independently correlated with CIMT too. An evaluation of SUA combined with other factors (such as age, SBP, FBG) may provide a more accurate prediction of CIMT thickening.

Stroke is a serious health hazard with high mortality and disability rates and has gradually become the leading cause of death in humans (30, 31). Approximately 5.5 million stroke-related deaths occur each year (32). Around 30% of patients with an initial onset of acute cardio-cerebrovascular disease exhibit no clinical symptoms (33). Most strokes are caused by arterial occlusion due to the formation of an embolus due to carotid plaque rupture (34). Changes in CIMT occur earlier than the formation of plaques during the development of carotid atherosclerosis. SUA acts as an antioxidant scavenger that can provide powerful free radical scavenging capacity in the plasma, so SUA offers certain benefits, but there is also evidence from animal models and epidemiological studies of an association between the increase in SUA concentration and adverse cardiovascular and cerebrovascular risk factors (35, 36). In a study in hospitalized older adults in Japan, CIMT and the prevalence of carotid atherosclerosis were shown to significantly increase with an increase in the uric acid quartile in both men and women without metabolic syndrome, which suggests that SUA was an independent risk factor for carotid atherosclerosis (37). Meanwhile a clinical trial in the United States reported that SUA levels were positively correlated with CIMT and associated with significant vascular stenosis in patients with ischemic stroke, which indicated that SUA played a key role in the pathogenesis of atherosclerosis and participates in the development of ischemic stroke (21). In China, a study by Song et al. in middle-aged and older Chinese adults also confirmed a linear relationship between SUA and CIMT (38). Indeed, the independent risk of cardiovascular disease in a general Italian population significantly increases when uric acid levels exceed 5.6 mg/dl (39). However, several studies have shown that the incidence of cardiovascular and cerebrovascular diseases does not increase in patients with hyperuricemia without gout (40, 41). According to National Health and Nutrition Examination Survey III, the relationship between SUA level and cardiovascular disease mortality was u-shaped (42). Both low and high uric acid levels may be independent risk factors for cardiovascular disease; moreover, uric acid levels over 7 mg/dl were an independent risk factor for stroke (42). Our study further confirmed SUA can be used as an independent risk factor for carotid atherosclerosis in Chinese people.

Mendelian randomized analysis showed that elevated levels of SUA lead to elevated blood pressure, which in turn, increased the risk of cardiovascular diseases, such as coronary heart disease and stroke (43). A study in Chinese patients with type 2 diabetes found that elevated SUA levels were strongly associated with carotid atherosclerosis (44). Diabetes is associated with an increased risk of atherosclerotic disease, and people with diabetes have a higher risk of stroke than those without diabetes (45, 46). Previous genome-wide studies have shown that impaired glucose tolerance is significantly associated with CIMT, and a genome-wide study on uric acid salt found that transcription factors involved in urate metabolism may be associated with various metabolic processes of synergy between adjustment, such as blood glucose and other cardiovascular risk factors, which may increase the likelihood of confusion regarding causality (47). We found that Age, SBP, FBG, and SUA played important roles in carotid atherosclerosis. However, because age is an uncontrollable factor for cardiovascular disease, more attention should be directed toward controllable factors, such as SBP, FBG, and SUA. FBG and SBP play crucial roles in the development of stroke (48–50), and our study demonstrated that the effect of SUA on carotid atherosclerosis should not be ignored. SUA, in combination with other factors, may serve as a specific model to help predict the incidence of CIMT thickening. Previous studies have shown that LDL-C causes atherosclerosis. However, we did not observe a correlation between LDL-C and CIMT. We speculate that this discrepancy is related to the administration of lipid-lowering drugs to some patients. Given the correlation between SUA level and CIMT, uric acid could be considered an independent risk factor for carotid arteriosclerosis as well as a potential intervention target. Several studies have shown that reducing uric acid by administering allopurinol, febuxostat, and other drug treatments can significantly improve local and systemic endothelial function, peripheral vasodilation capacity, and blood flow and delay the progression of CIMT (51–53). However, determining its impact on cardiovascular and cerebrovascular diseases requires prospective studies to be conducted using reliable clinical endpoints.

Uric acid is a damage-related molecule that is released during tissue ischemia and cell death. When uric acid crystallizes, it activates nucleotide-binding oligomerization domain-like receptor protein 3 (NLRP-3) inflammasomes, which leads to apoptosis-associated speck-like protein-containing speck formations, caspase-1 activation, and interleukin (IL)-1β production. NLRP-3 and IL-1β are associated with plaque instability and atherogenesis. Urate crystals trigger a series of inflammatory reactions by activating the NLRP-3 pathway, promoting an increase in CIMT, which leads to plaque formation and ultimately, cardiovascular and cerebrovascular events (54, 55). However, the mechanism of increased blood uric acid leading to CIMT remains controversial. In general, researchers worldwide agree that blood uric acid leads to the development of atherosclerosis *via* the following mechanisms: 1) elevated blood uric acid level promotes low-density lipoprotein oxidation and lipid peroxidation, which in turn, contributes to the development of atherosclerosis (56); 2) when blood uric acid concentration increases, arteriosclerotic uric acid microcrystals precipitate easily, are deposited in the vascular intima, and cause local inflammatory reactions, which directly damages vascular endothelial cells, eventually resulting in lipid deposition (57); 3) an increase in blood uric acid is often accompanied by the aggregation of reactive oxygen species, such as oxygen free radicals and hydrogen peroxide, which leads to vascular inflammatory reactions (58); 4) urate, as an inflammatory substance, directly promotes platelet aggregation and thrombosis, whereas cytokines released by platelets cause vascular smooth muscle hyperplasia (51); and 5) elevated blood uric acid level has been linked to obesity, insulin resistance, dyslipidemia, and hypertension, all of which are associated with an increased risk of cardiovascular disease. Drop uric acid treatment is the main method used to promote uric acid excretion, and studies have shown that drop uric acid treatment can reduce blood pressure. Patients with high uric acid hematic disease who have high blood pressure, especially young patients without a long history of high blood pressure; otherwise, high uric acid hematic disease patients with a prolonged period of high blood pressure eventually develop chronic kidney disease due to uric acid excretion dysfunction. This, in turn, forms a vicious cycle of hyperuricemia and subsequently triggers or aggravates cardiovascular disease (59). Moreover, several studies have confirmed that SUA-lowering therapy can improve outcomes of patients with a cardiovascular or cerebrovascular disease to a certain extent (60).

Our study had several limitations. First, the participants in the study were recruited from only two hospitals, and the number of analyzed cases in the study was small. Thus, the study population may not represent the current situation of patients with hyperuricemia across China. Moreover, the study lacked reliable comprehensiveness to clarify certain relationships. Further prospective studies with larger and more representative samples are needed to confirm the association between SUA level and cerebrovascular disease. Second, we only evaluated FBG levels of the participants, and postprandial blood glucose and hemoglobin A1c levels were not evaluated, which may impact the identification of glucose metabolic conditions. Finally, whether uric acid-lowering treatment influences the outcomes of patients with cardiovascular and cerebrovascular diseases requires further study.

Our findings suggested that SUA was an independent risk factor for carotid atherosclerosis and that an elevated SUA level promoted thickening of the carotid intima depending on FBG patterns. SUA, combined with age, SBP, FBG, could be used as a specific model to help predict the incidence of CIMT thickening. An in-depth study of the specific underlying mechanism would be valuable for the early prevention of carotid vascular diseases.

## Data Availability Statement

The raw data supporting the conclusions of this article will be made available by the authors, without undue reservation.

## Ethics Statement

The studies involving human participants were reviewed and approved by the Ethics Committee of the China-Japan Union Hospital of Jilin University (2020032509) and the Ethics Committee of the First Hospital of Jilin University (2020-420). The patients/participants provided their written informed consent to participate in this study. Written informed consent was obtained from the individual(s) for the publication of any potentially identifiable images or data included in this article.

## Author Contributions

YG and BX were involved in data collection and article writing; RL designed the project and was responsible for article revisions; YY and MZ were involved in measuring CIMT; TY and YG were responsible for literature retrieval; QZ and JS were responsible for chart construction. All authors contributed to the article and approved the submitted version.

## Funding

This work was supported by grants from the Department of Science and Technology of Jilin Province (20210204217YY), the Interdisciplinary Innovation Project of the First Hospital of Jilin University (JDYYJCHX2020014), and the Scientific Research Project of the Jilin Provincial Department of Education (JJKH20211162KJ).

## Conflict of Interest

The authors declare that the research was conducted in the absence of any commercial or financial relationships that could be construed as a potential conflict of interest.

## Publisher’s Note

All claims expressed in this article are solely those of the authors and do not necessarily represent those of their affiliated organizations, or those of the publisher, the editors and the reviewers. Any product that may be evaluated in this article, or claim that may be made by its manufacturer, is not guaranteed or endorsed by the publisher.
